# A Potential Role for Fructosamine-3-Kinase in Cataract Treatment

**DOI:** 10.3390/ijms22083841

**Published:** 2021-04-07

**Authors:** Sander De Bruyne, Loes van Schie, Jonas Himpe, Filip De Somer, Inge Everaert, Wim Derave, Caroline Van den Broecke, Manon Huizing, Nezahat Bostan, Marijn Speeckaert, Nico Callewaert, Elisabeth Van Aken, Joris R. Delanghe

**Affiliations:** 1 Department of Diagnostic Sciences, Ghent University, 9000 Ghent, Belgium; sanderR.debruyne@ugent.be (S.D.B.); jonas.himpe@ugent.be (J.H.); 2 VIB-UGent Center for Medical Biotechnology, VIB, 9052 Ghent, Belgium; loes.vanschie@vib-ugent.be (L.v.S.); nico.callewaert@vib-ugent.be (N.C.); 3 Department of Biochemistry and Microbiology, Ghent University, 9052 Ghent, Belgium; 4 Department of Human Structure and Repair, 9000 Ghent, Belgium; Filip.DeSomer@ugent.be; 5 Department of Movement and Sport Sciences, Ghent University, 9000 Ghent, Belgium; Inge.Everaert@UGent.be (I.E.); wim.derave@ugent.be (W.D.); 6 Department of Pathology, Ghent University Hospital, 9000 Ghent, Belgium; caroline.vandenbroecke@azstlucas.be; 7 Antwerp Biobank, Antwerp University Hospital, 2650 Antwerp, Belgium; manon.huizing@uza.be (M.H.); nezahat.bostan@uza.be (N.B.); 8 Department of Internal Medicine and Pediatrics, Ghent University, 9000 Ghent, Belgium; marijn.speeckaert@ugent.be; 9 Department of Head and Skin, Ghent University, 9000 Ghent, Belgium

**Keywords:** advanced glycation end products, cataract, deglycation, fructosamine-3-kinase, therapeutics

## Abstract

Cataracts are the major cause of blindness worldwide, largely resulting from aging and diabetes mellitus. Advanced glycation end products (AGEs) have been identified as major contributors in cataract formation because they alter lens protein structure and stability and induce covalent cross-linking, aggregation, and insolubilization of lens crystallins. We investigated the potential of the deglycating enzyme fructosamine-3-kinase (FN3K) in the disruption of AGEs in cataractous lenses. Macroscopic changes of equine lenses were evaluated after ex vivo intravitreal FN3K injection. The mechanical properties of an equine lens pair were evaluated after treatment with saline and FN3K. AGE-type autofluorescence (AF) was measured to assess the time-dependent effects of FN3K on glycolaldehyde-induced AGE-modified porcine lens fragments and to evaluate its actions on intact lenses after in vivo intravitreal FN3K injection of murine eyes. A potential immune response after injection was evaluated by analysis of IL-2, TNFα, and IFNγ using an ELISA kit. Dose- and time-dependent AF kinetics were analyzed on pooled human lens fragments. Furthermore, AF measurements and a time-lapse of macroscopic changes were performed on intact cataractous human eye lenses after incubation with an FN3K solution. At last, AF measurements were performed on cataractous human eyes after crossover topical treatment with either saline- or FN3K-containing drops. While the lenses of the equine FN3K-treated eyes appeared to be clear, the saline-treated lenses had a yellowish-brown color. Following FN3K treatment, color restoration could be observed within 30 min. The extension rate of the equine FN3K-treated lens was more than twice the extension rate of the saline-treated lens. FN3K treatment induced significant time-dependent decreases in AGE-related AF values in the AGE-modified porcine lens fragments. Furthermore, in vivo intravitreal FN3K injection of murine eyes significantly reduced AF values of the lenses. Treatment did not provoke a systemic immune response in mice. AF kinetics of FN3K-treated cataractous human lens suspensions revealed dose- and time-dependent decreases. Incubation of cataractous human eye lenses with FN3K resulted in a macroscopic lighter color of the cortex and a decrease in AF values. At last, crossover topical treatment of intact human eyes revealed a decrease in AF values during FN3K treatment, while showing no notable changes with saline. Our study suggests, for the first time, a potential additional role of FN3K as an alternative treatment for AGE-related cataracts.

## 1. Introduction

**Figure 1:**
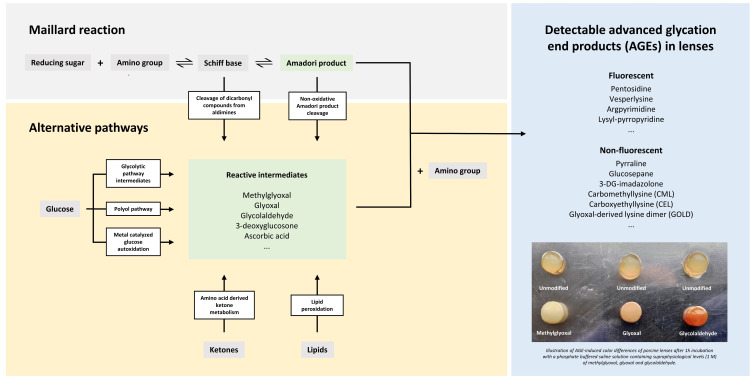
Advanced glycation end products (AGEs) are synthesized endogenously via the Maillard reaction and several alternative pathways [6]. In the early steps of the Maillard reaction, an iminine intermediate (Schiff base) is formed by the reaction of a reducing sugar (e.g., glucose) with a protein-bound amine or free amino acid. The labile Schiff base can produce highly reactive dicarbonyls (e.g., methylglyoxal) or rearrange into stable Amadori products. In the advanced steps of the Maillard reaction, the Amadori products undergo complex rearrangements, cleavage and covalent binding reactions, which eventually give rise to the generation of AGEs, which are stable adducts and cross-links of proteins [6,7]. Highly reactive carbonyls can also be generated from glycolytic pathway intermediates, the polyol pathway, glucose autoxidation, ketones generated by the breakdown of amino acids and lipid peroxidation. Those carbonyl compounds initiate the process of advanced glycation directly, eventually leading to the synthesis of a wide range of fluorescent (e.g., pentosidine) and non-fluorescent AGEs (e.g., glucosepane), which can be found in human tissues including eye lenses [6].

Cataracts are a global public health problem with a rising prevalence in both industrialized and third-world countries [1]. It is the major cause of blindness worldwide, affecting about 65.2 million people [2] and one of the principally targeted avoidable eye conditions in the VISION 2020 project for the World Health Organization [3]. While there is a wide range of potential causes, aging is considered as the most important risk factor with excessive exposure to ultraviolet light and related free radical damage of crystallins being principal pathogenic components [4]. Moreover, due to a very low protein turnover, crystallins belong to the longest-lived human proteins and are prone to progressive glycation with age. α-, β- and γ-crystallins make up >90% of the total dry mass of the lens [5]. In the process of protein glycation, reducing sugars and carbonyls react with free amino groups, forming adducts that can then rearrange and react further, eventually leading to protein cross-links. This process in which advanced glycation end products (AGEs) are formed is generally known as the Maillard reaction, a very complex and quite incompletely understood non-enzymatic set of reactions [4]. An overview of the steps involved in the endogenous formation of AGEs can be found in Figure 1.

The Maillard theory with progressive accumulation of AGEs may contribute not only to general aging, but also to the pathology of metabolic diseases, such as diabetes and atherosclerosis, and neurodegenerative diseases [8,9,10]. In the lens, AGEs have been characterized during normal aging as well as in a diabetic context [9]. Non-enzymatic glycation of lens proteins is a major factor responsible for cataract formation by altering lens protein structure and stability and by inducing covalent cross-linking, aggregation and insolubilization of lens crystallins [11]. Accumulation of AGEs is more substantial in diabetics as the elevated glucose concentration accelerates the production of AGEs [12]. In aging and diabetes, increased concentrations of dicarbonyl compounds (e.g., methylglyoxal and glyoxal) also result in AGE cross-links on α-crystallins with a consequent reduction of chaperone activity, enhanced αβ-crystallin content, and the formation of dense aggregates [4]. Concomitantly, prevalence of cataracts in diabetics is five-fold higher than among non-diabetics [12]. AGEs also contribute to cataracts through their cytotoxic effect on epithelial lens cells through the induction of apoptosis in a NF-kappa b-dependent manner. Furthermore, AGEs accumulate in aging human lens capsules, suggesting a role in the transforming growth factor beta-2-mediated fibrosis of epithelial lens cells during posterior capsule opacification [13].

As current surgical cataract treatment by phacoemulsification is relatively expensive and the number of practicing ophthalmologists is small in developing countries, there is a growing demand for affordable cataract treatment options accessible to a large number of patients. AGE-inhibiting or disrupting compounds may have efficacy in the prevention and treatment of AGE-related cataract formation. Present knowledge suggests that FN3K is a part of the natural cellular repair mechanisms that control non-enzymatic glycation of proteins and is more active in tissues with a long half-life (e.g., brain, erythrocyte, eye lens) [14,15,16]. The FN3K enzyme is known to phosphorylate both free and protein-bound fructosamines (Amadori rearrangement products) on the third carbon of their sugar moiety making them unstable, which leads to their detachment from proteins with concomitant regeneration of the unglycated amine [17]. This process eventually prevents the formation of AGEs. Lower AGE levels have been reported in populations with higher concentration and activity levels of FN3K [18], and a protective role of the enzyme has recently been documented against the onset of vascular complications in the diabetic population [19]. Moreover, FN3K has successfully been used in the deglycation of several tissue types such as heart valves [20], nails [21,22] and kidney sections [23].

Next to the classical view of FN3K being an intracellular enzyme that has primary (low molecular mass) glycation products as a unique substrate, recent research suggested an additional potential role of FN3K in the direct disruption of retinal AGEs [24]. However, it is still unexplored whether FN3K treatment would also result in the disruption of AGEs and cross-linked structures of cataractous lenses. Therefore, we studied the effects of FN3K treatment on cataractous lenses of both animal and human origin, mainly by assessing the effects of FN3K treatment on the amount of Maillard-type autofluorescence.

## 2. Results

### 2.1. In Vitro Kinase Activity of FN3K, but Not FN3K–K41L, Is Dose-Dependent

**Figure 2:**
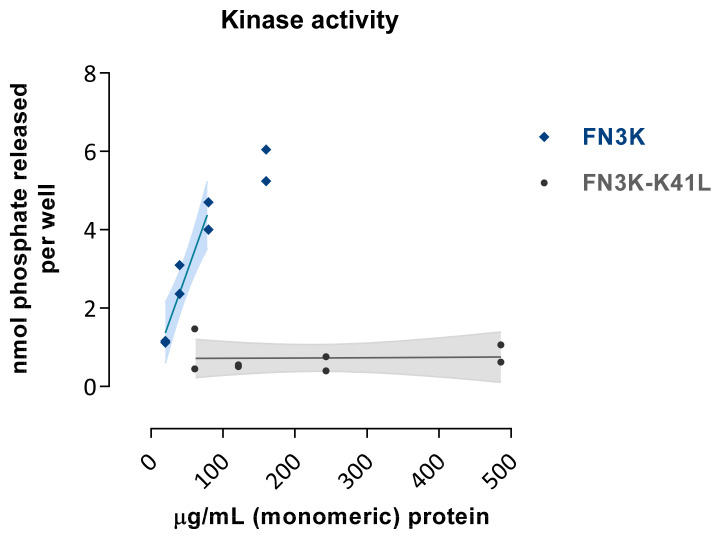
Kinase activity of FN3K and the inactive mutant enzyme FN3K–K41L as assessed by the amount of phosphate released per well (nmol) using a 1-deoxy-1-morpholino-D-fructose (DMF) acceptor substrate at increasing enzymatic concentration levels (µg/mL). Data are depicted as single data points with their fitted regression line in the linear range and 95% confidence interval.

The kinase activity of FN3K was dose-dependent, with 108 pmol µg^−1^ min^−1^ phosphate released (derived from regression in the linear range), whereas FN3K–K41L resulted in minimal phosphate release (0.2 pmol µg^−1^ min^−1^, Figure 2). However, we do not know whether this lack of activity is purely due to the K41L mutation in the catalytic center, or to misconformation or aggregation. This limitation should be taken into account when using FN3K–K41L as a negative control sample for FN3K in in vivo functional experiments.

### 2.2. Ex Vivo Intravitreal FN3K Treatment of Equine Lenses

**Figure 3:**
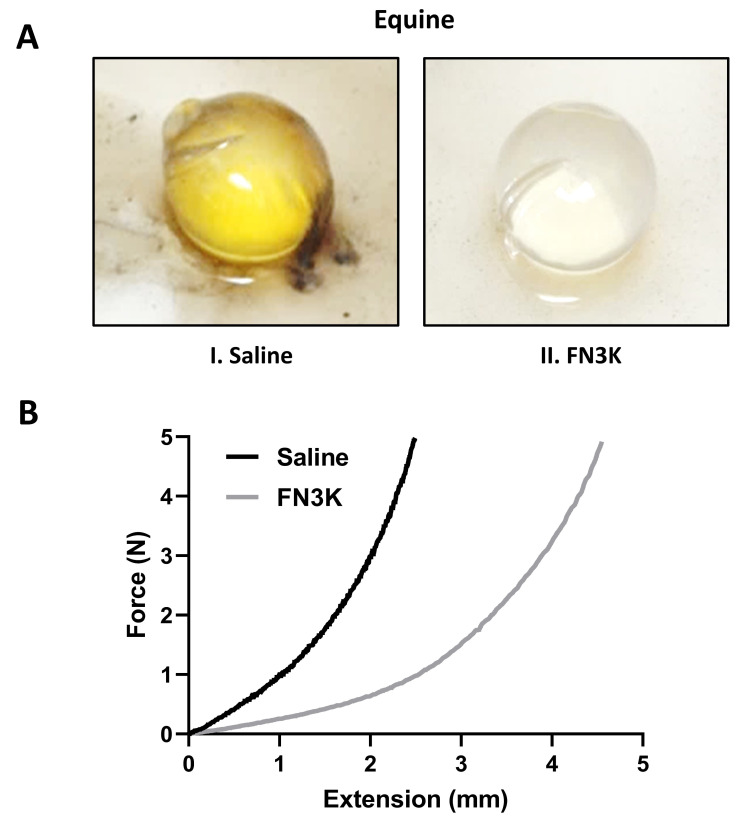
(**A**) Macroscopic aspect of a representative ex vivo saline-(I) and fructosamine-3-kinase (FN3K) treated (II) lens originating from equine eye pairs. (**B**) Extension rate of lenses originating from an equine eye pair treated with either saline or FN3K. N, newton.

While the lenses of the equine FN3K-treated eyes (*n* = 3) appeared to be clear, the saline-treated lenses (*n* = 3) had a yellowish-brown color. Following FN3K treatment, complete color restoration could be observed within 30 min (Figure 3A). For a force up to 5 N, the extension rate of one of the equine FN3K-treated lenses appeared to be more than twice the extension rate of the saline-treated lens (Figure 3B).

### 2.3. FN3K Treatment of AGE-Modified Porcine Lens Fragments Reduces Maillard-Type Autofluorescence

**Figure 4:**
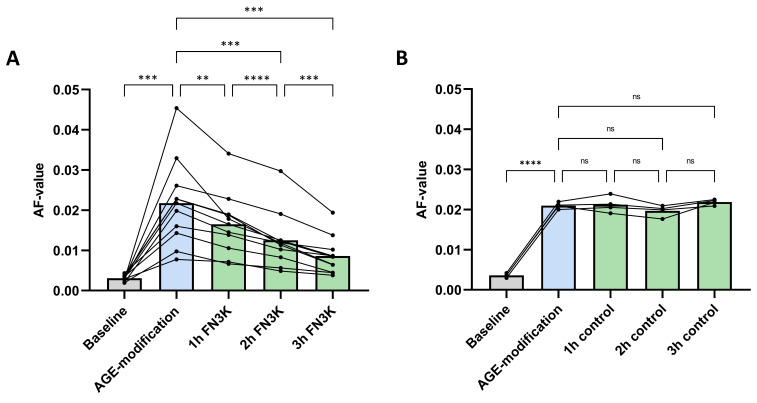
(**A**) Autofluorescence (AF) values of glycolaldehyde-induced advanced glycation end product (AGE)-modified porcine lens fragments after fructosamine-3-kinase (FN3K, *n* = 11) and (**B**) control (*n* = 4) treatment with the cofactors ATP and MgCl_2_ solely. The bars represent the mean. **** *p* < 0.0001; *** *p* < 0.001; ** *p* < 0.01; ns, non-significant.

Figure 4A shows the autofluorescence (AF) values of the porcine lens fragments (*n* = 11) at baseline, after AGE-modification and after FN3K treatment (1 h, 2 h and 3 h incubation). Repeated measures ANOVA revealed significant changes between the different treatment groups (*p* < 0.0001). AGE-related AF values significantly increased in all the porcine lens fragments after incubation with 25-mM glycolaldehyde (AF value 0.022 ± 0.011) compared to baseline levels (AF value 0.0031 ± 0.00072, *p* = 0.0002). Subsequently, FN3K treatment induced significant and ongoing decreases of AF values after 1 h (AF value 0.017 ± 0.0077, −22.7%, *p* = 0.0021), 2 h (AF value 0.013 ± 0.0069, −40.9%, *p* = 0.0002) and 3 h (AF value 0.0086 ± 0.0046, −60.9%, *p* = 0.0002) compared to the levels obtained after glycolaldehyde modification. No notable changes were observed after control treatment (cofactors ATP and MgCl_2_ solely) of the AGE-modified lens fragments (*n* = 4, Figure 4B).

### 2.4. In Vivo Intravitreal FN3K Injection of Murine Eyes Reduces Maillard-Type Autofluorescence

**Figure 5:**
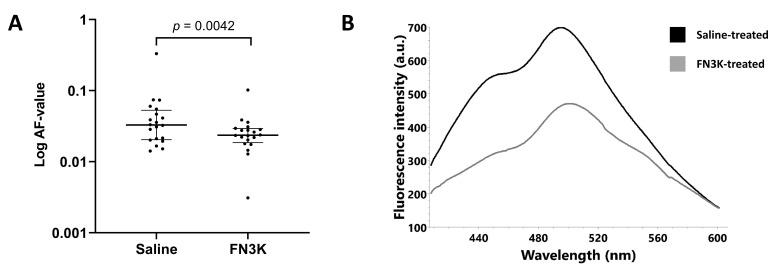
(**A**) Autofluorescence (AF) values of the enucleated lenses of *ob/ob* (*n* = 20) mice after in vivo intravitreal injection with saline (left eye) or FN3K (right eye). Data are displayed as the median and interquartile range (log scale). (**B**) Median fluorescence emission spectra (400–620 nm range) of the murine lenses after in vivo saline (black line) or FN3K (grey line) treatment. A.u., arbitrary units; nm, nanometer.

Concerning the AF values, the Wilcoxon test revealed that the median of the differences between saline-(*n* = 20, median 0.033, IQR 0.021–0.051) and FN3K-(*n* = 20, median 0.024, IQR 0.019–0.029) treated lenses was statistically different (*p* = 0.0042, Figure 5A). Figure 5B illustrates the median fluorescence emission spectra (400–620 nm range) for both treatment conditions.

**Figure 6:**
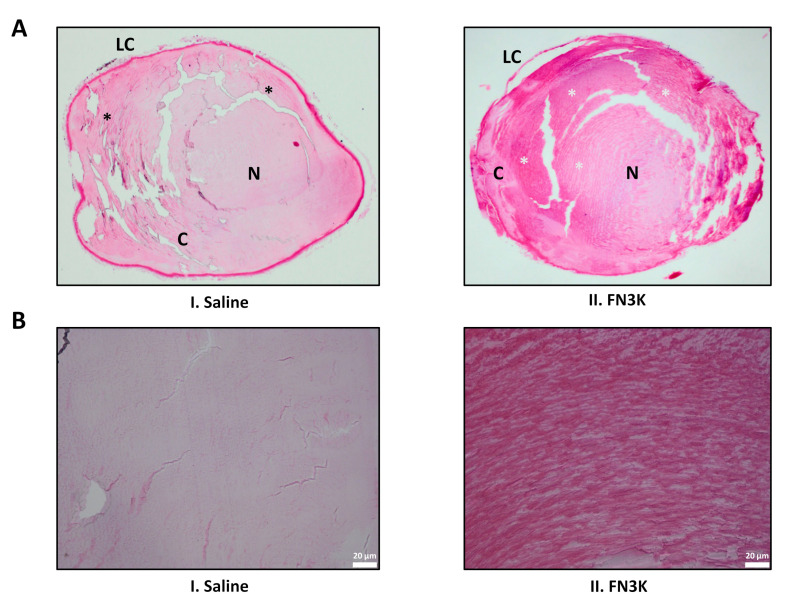
(**A**) Hematoxylin and eosin-stained sections of a saline (I) and FN3K treated (II) lens originating from the same *ob/ob* mouse. Disorganized and organized zones of lens fibers are indicated with black and white asterisks, respectively. C, cortex; LC, lens capsule; N, nucleus. (**B**) Close-up on the cortical lens fibers of the saline (I) and FN3K treated (II) lens.

Figure 6A displays the hematoxylin and eosin (HE) stained tissue section of a saline-treated lens and its FN3K-treated counterpart. In both lenses, the lens capsule was present. In the cortex of the saline-treated eye, the parallel arrangement of the lens fibers was disturbed with a disorganized aspect of the subcapsular layer. While the cortex of the FN3K-treated eye was also characterized by disorganized zones of lens fibers, multiple subcortical zones showed the presence of organized lens fibers. Furthermore, lenses of the FN3K-treated mice eyes appeared to be more spherical in shape compared to the more flattened lenses of saline-treated eyes. Figure 6B shows a close-up on the cortical lens fibers. In the FN3K-treated lens, fibers are tightly packed with little intercellular space and are concentrically arranged. In contrast, the architecture of the cortical lens fibers in the control-treated counterpart is disturbed with the presence of vacuoles and loss of parallel organization.

### 2.5. FN3K Treatment Does Not Provoke a Systemic Immune Response in Mice

In all non-treated mice (*n* = 5) serum levels of IL-2, TNFα and IFNγ were below detection limits (2 pg/mL, 8 pg/mL and 16 pg/mL, respectively). In a large majority of FN3K-treated mice (45 out of 55), levels of these 3 cytokines were below detection limits as well. In several serum samples (10 out of 55), one of the three cytokines tested for was upregulated. However, cytokine levels were not consistently high within treatment groups and in general were very low (below 120 pg/mL). From this, we could conclude that intravitreal injection of a therapeutic FN3K mixture did not consistently provoke a systemic immune response. ELISA results are enclosed in Appendix A.

### 2.6. Gel Filtration of Human Cataractous Lens Fractions Reveals Breakdown Products after FN3K Treatment

**Figure 7:**
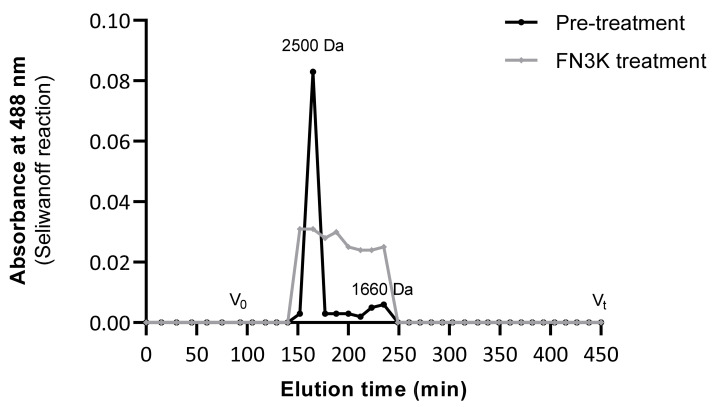
Gel filtration patterns (Sephadex^®^ G-25 Fine resin) of urea-soluble lens fractions before and after FN3K treatment showing the absorbance at 488 nm (Seliwanoff reaction) of high-molecular mass fructose containing compounds (AGEs) as a function of the elution time. V_0_ (>5 kDa) indicates the void volume, Vt (<500 Da) the terminal volume.

Comparison of gel filtration patterns of urea-soluble pooled human lens fractions before and after FN3K treatment (Figure 7) showed a reduction of the mean molecular mass of the fructose containing compounds. In the untreated lens, a peak corresponding to a molecular mass of ± 2500 Da and a second minor peak corresponding to 1660 Da were observed, which indicates the presence of high-molecular mass structures such as cross-linked AGEs. In the FN3K treated lens fractions, a series of breakdown products with a molecular mass ranging between 1500 and 2500 Da were detected. This strengthens our hypothesis that the enzyme is not only active against low-molecular mass glycation products, but also possesses activity against high-molecular mass compounds (AGEs).

### 2.7. Autofluorescence Kinetics of FN3K Treatment on Cataractous Human Lens Suspensions

**Figure 8:**
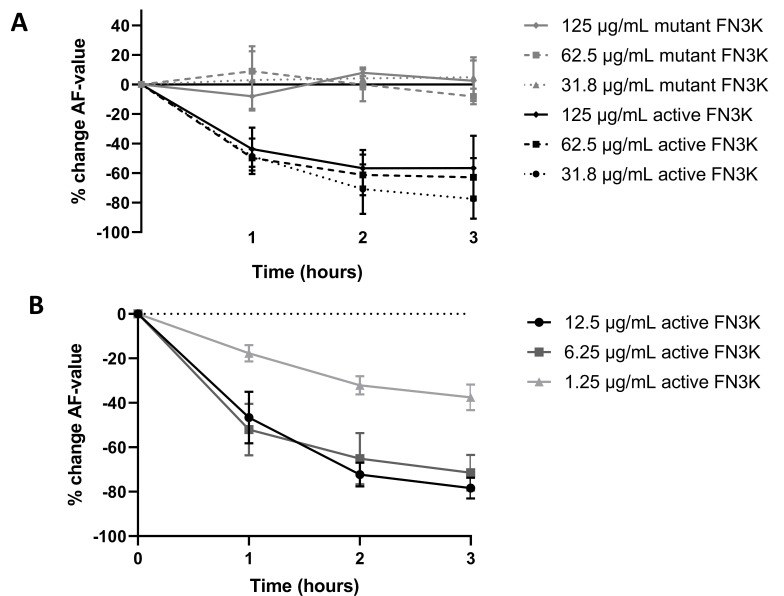
(**A**) Dose- and time-dependent changes (%) in autofluorescence (AF) values after treatment of a cataractous human lens suspension with active and mutant fructosamine-3-kinase (FN3K) in a higher concentration range: 125 µg/mL, 62.5 µg/mL and 31.8 µg/mL. (**B**) Dose- and time-dependent changes (%) in AF values after treatment with active FN3K in a lower concentration range (12.5 µg/mL, 6.25 µg/mL and 1.25 µg/mL). Data are presented as the mean of *in triplo* performed experiments with the standard error of the mean.

Figure 8A shows the mean change (%) of AF values compared to baseline levels for cataractous human lens suspensions in function of incubation time at a higher concentration range of both the active and mutant FN3K enzyme: 125 µg/mL, 62.5 µg/mL and 31.8 µg/mL. While only small or negligible changes of AF values were noticed in the case of the mutant enzyme, remarkable decreases were observed for the active enzyme. Similar kinetics were found at all concentrations levels. The most pronounced decrease of AF values was observed after 1 h incubation (43.6–49.7% decrease compared to baseline). While after the second hour some additional progression of the chemical reaction was observed (56.7–70.8% decrease compared to baseline), AF values remained rather stable after the third hour (56.6–77.4% decrease compared to baseline). The latter suggests the completion of the reaction process at the end of the experiment. Figure 8B shows the mean change (%) of AF values compared to baseline levels in function of incubation time at a lower concentration range of active FN3K (12.5 µg/mL, 6.25 µg/mL and 1.25 µg/mL). In contrast to the experiment performed in the higher range, a more notable time- and dose-dependent effect was observed. While similar kinetics were noted for lens suspensions incubated with 12.5 µg/mL and 6.25 µg/mL of active FN3K, the reaction process appeared to be explicitly slower for suspensions incubated with 1.25 µg/mL. At all concentration levels, an additional decrease of AF values was seen after 3 h of incubation which suggests an ongoing reaction process. Additional control experiments revealed that neither the added ATP nor the ADP released by the active enzyme, was responsible for the observed decreases in AF after FN3K treatment (Appendix B).

### 2.8. Incubation of Cataractous Human Eye Lenses with FN3K

**Figure 9:**
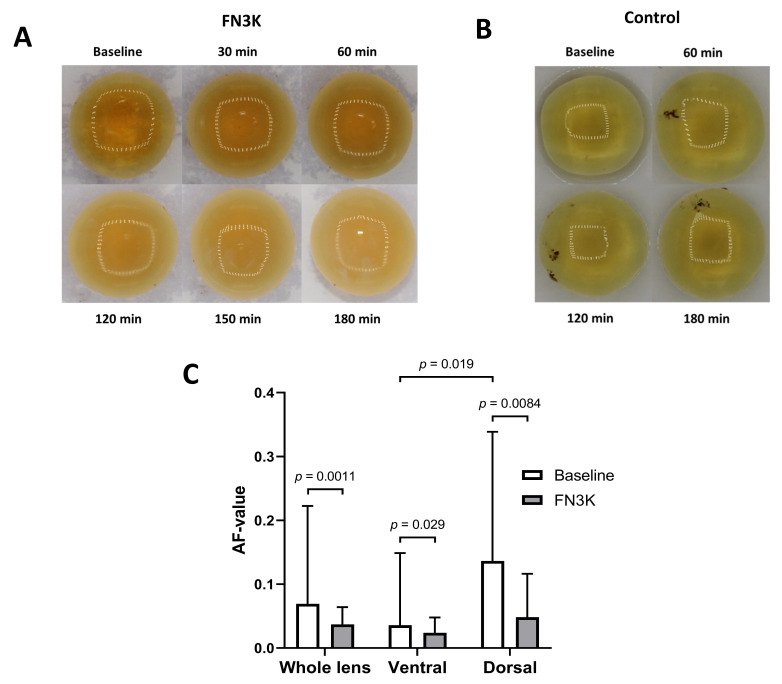
Time-lapse of the macroscopic changes observed after (**A**) ex vivo fructosamine-3-kinase (FN3K) treatment and (**B**) saline treatment of cataractous human eye lenses originating from two different patients. After 30 min, an improvement of the color of the cortex and homogeneity could be observed. During a follow-up in the subsequent 2.5 h, the light exposure was changed, which hampered the interpretation of color intensities in the pictures. However, independent of light exposure differences, a further amelioration of the color of the cortex and homogeneity could be observed. No remarkable macroscopic changes could be observed in the case of the saline-treated lens. (**C**) Autofluorescence (AF) values displayed as the median and interquartile range of a cataractous human eye lens at 30 different measurement locations at baseline and after FN3K treatment for the ventral, dorsal, and whole lens surface.

Figure 9A shows a time-lapse of the macroscopic changes observed after FN3K treatment of a cataractous human eye lens. After 30 min, an improvement of the color of the cortex and homogeneity could be observed. During a follow-up in the subsequent 2.5 h, the light exposure was changed, which hampered the interpretation of the color intensities in the pictures. However, independent of light exposure differences, a further amelioration of the color of the cortex and homogeneity could be observed. Some minor additional improvements were found at the end of the monitoring period (after 3 h). No remarkable color changes could be observed in the case of the saline-treated lens (Figure 9B). For another cataractous human eye lens, a Mann–Whitney U test on spectral measurements of the whole lens revealed a statistically significant decrease of the AF value after ex vivo incubation of the intact eye lens with FN3K (median 0.037, IQR 0.018–0.063) compared to baseline levels (median 0.069, IQR 0.033–0.20, *p* = 0.0011). AF values appeared to be significantly lower at the ventral surface of the lens (median 0.036, IQR 0.029–0.13) compared to the dorsal surface (median 0.14, IQR 0.057–0.34, *p* = 0.019). Furthermore, AF values at the dorsal surface of the lens showed a stronger decrease after FN3K treatment (median 0.048, IQR 0.022–0.11, *p* = 0.0084) compared to the ventral surface (median 0.024, IQR 0.017–0.047, *p* = 0.029, Figure 9C).

### 2.9. Topical Treatment of Post-Mortem Human Eyes

For the left eye of a 78-year-old cataract patient, treatment with saline-containing drops did not reveal noteworthy changes to the AF value. However, after crossover treatment with FN3K, a decrease of 53.9% was observed. For the right eye, a rather small decrease of 16.4% was noted after FN3K treatment, while treatment with saline did not reveal notable changes. The smaller decrease in the right eye can potentially be attributed to the fact that the baseline AF value of the patient’s left eye (AF value = 0.0166) appeared to be higher than that of the right eye (AF value = 0.0101), indicating a higher content of fluorescent AGEs and therefore a higher level of potential substrates. The results are summarized in Table 1.

## 3. Discussion

Our first preliminary animal experiments showed a color restoration of equine cataractous lenses after ex vivo intravitreal FN3K injection. Furthermore, the extension rate of an equine FN3K-treated lens more than doubled compared to its control-treated counterpart. It is already well known that, according to the classical view as illustrated in Section 2.1, the FN3K enzyme removes ketoamines and consequently prevents the production of AGEs [18]. However, our results also indicate a potential additional role of recombinant FN3K and its cofactors in the breakdown of high molecular mass compounds (AGEs) in cataractous lenses.

Both in vitro FN3K treatment of AGE-modified porcine lens fragments as well as in vivo intravitreal FN3K injection of murine eyes showed a significant reduction of Maillard-type autofluorescence. The latter without provoking a systemic immune response. We also observed a difference in lens geometry between saline- and FN3K-treated murine lenses. It is known that the glycation-induced aggregation and covalent crosslinking of lens crystallins can result in a decrease of natural elasticity and an increase in stiffness [25,26]. Normally the lens capsule together with the cortex, when not under tension of the zonules, causes the lens to assume a more rounded shape [27]. On the histological sections of the murine eye pair, the lens capsule was still present and intact in the lenses of both the FN3K- and saline-treated eyes. Consequently, FN3K did not only decrease the amount of Maillard-type autofluorescence, but also changed geometry in the cortex fibers, which strengthens our hypothesis that the process of non-enzymatic glycation of proteins, such as crystallins can be reversed. Several studies have demonstrated that Maillard reactions by sugars can induce a decrease in the chaperone function of α-crystallins [28,29,30]. Based on our results, we could hypothesize a potential role of FN3K in the restoration of α-crystallin chaperone activity. Positive modulation of α-crystallin chaperone activity has already been recognized as a possible evading strategy for cataract progression. Estimations have been made that a 10-year delay in cataract incidence can almost halve the number of cataract surgeries [31].

Similar results were obtained on human material. Gel filtration patterns of urea-soluble pooled human lens fractions revealed the presence of high molecular mass compounds, which broke down after FN3K treatment. AF kinetics on cataractous human lens suspensions revealed a dose- and time-dependent effect of treatment with the active FN3K enzyme, while showing no notable changes for the mutant enzyme. Besides, our data revealed a significant decrease of AF values after incubation of a cataractous human eye lens with FN3K and a lighter color of the cortex of another cataractous lens. At last, crossover topical treatment of post-mortem human eyes with saline and FN3K containing drops revealed a decrease of AF values when applying FN3K, while only small or negligible changes were noted after topical treatment with saline drops.

In 2010, one in three blind people became blind due to cataracts, and one of six visually impaired people became visually impaired due to cataracts, over 90% of whom live in low- and middle-income countries [32]. Cost, an insufficient number of ophthalmologists, and low government funding remain significant barriers to relieve the growing cataract burden by surgery alone. In addition to these, postoperative complications (e.g., posterior capsular opacification, endophthalmitis and uncorrected residual refractive error) can occur [33]. As a result, cataracts remains a major public health problem, especially in low- and middle- income countries, and there is a search for a pharmacological intervention that will restore the transparency of the lens. Our preliminary data might be promising for treating cataracts in a more cost-effective way, especially in developing countries where the need for an affordable treatment option rises year by year as people grow older and the prevalence of diabetes mellitus rises. Whereas the pathogenic role of AGEs in the development of cataracts has been well characterized at the molecular level in both normal aging as well as in the diabetic context [9], the unique structure of the lens is also vulnerable to a wide range of other factors: age-related (e.g., carbamoylation, deamidation, thiolation, proteolysis, antioxidant loss, free-radical scavenging) and non-age-related (e.g., genetics, trauma, uveitis, scleritis, radiation and toxic factors) [34,35]. Therefore, it is rather evident that FN3K treatment will not be able to provide an answer to all types of cataracts. Only a subgroup of cataract patients are expected to benefit from the potential therapeutic effects of FN3K.

Furthermore, while FN3K appears to be active against autofluorescent AGEs, we currently do not know its potential effect on non-autofluorescent AGEs. Albeit commercial antibody-based staining protocols for AGEs are available, their applicability is rather limited since AGEs represent a heterogeneous group of compounds and the exact targets of the antibodies have not yet been established [24]. So far, dozens of AGEs have been reported in vivo, with most of them being present at increased levels in cataractous lenses [13]. As an example, the non-fluorescent AGE glucosepane is known to be present to a higher degree in senile cataractous lenses compared to age-matched non-cataractous lenses [30,36]. However, the occurrence of specific AGEs can also depend on the type of cataract. Whereas higher amounts of the fluorescent AGE pentosidine have been found in diabetic cataracts compared to senile cataracts, levels of the non-fluorescent AGE N^ε^-carboxymethyllysine have been reported to be rather comparable between the two cataract types [37]. While ongoing diffusion experiments are being conducted to investigate the ocular and lens penetration of FN3K for intravitreal injections as well as for topical eye drops, we could hypothesize a potential role for the lens microcirculation system in the delivery of FN3K molecules within the lens [38,39]. Nevertheless, with aging, the development of a barrier to the transport of molecules within the lens has been reported [40,41]. Such a barrier could complicate an optimal influx of the enzyme within the lens interior. However, we could hypothesize that the destruction of cross-linked AGEs by FN3K has the ability to reduce the viscosity of the diffusion medium (the lens), thereby facilitating its transport within it.

Our study is hampered by several limitations. First of all, experiments in this study have only been performed on human material in vitro or ex vivo. Human clinical trials are indispensable for assessing the clinical validity of our findings. Furthermore, mice were treated with only one injection of FN3K. It can be expected that in the in vivo situation multiple treatment rounds will be necessary to obtain the most optimal results. Besides, it might as well be that after time in humans, crosslinking of the βγ-crystallins recurs and treatment should be repeated. At last, the power of our study is hampered by a low number of intact human eye lenses.

Overall, it can be concluded that the FN3K enzyme represents a potential additional treatment option for AGE-related cataract, which can be of special interest to developing countries. While our preliminary results are in need of validation on larger sample sizes and need to be confirmed by human clinical trials, our findings pave the way for future research.

## 4. Materials and Methods

### 4.1. Production of Active and Mutant Fructosamine-3-Kinase

#### 4.1.1. Construct Design

A DNA construct encoding human FN3K (UniProt Q9H479) flanked by an N-terminal His-tag with a caspase-cleavable D–E–V–D site was codon-optimized for *P. pastoris* (GeneArt, Regensburg, Bavaria, Germany) and cloned into the pKai61 vector [42] for intracellular expression. In light of preclinical experiments with FN3K, a catalytically inactive mutant of the protein would be a valuable asset as a suitable negative control. We, therefore, produced a mutant human FN3K carrying a K41L substitution (FN3K–K41L) in the putative F–V–K catalytic triad. Plasmids encoding codon-optimized FN3K–K41L flanked by an N-terminal His-tag with a caspase-cleavable D–E–V–D site were created by introducing synthetic DNA fragments into a Golden Gate assembly-based modular cloning system [43]. Electrocompetent cells, prepared according to a condensed protocol [44], were transformed with the *PmeI*-linearized vectors.

#### 4.1.2. Protein Production and Purification

*P. pastoris* cultures of up to 2 L were grown in baffled shake flasks with a maximum of 250 mL culture per 2 L flask. Cells were grown for 48 h at 28 °C in a BMGY medium (100 mM potassium phosphate pH 6, 2%w/v peptone, 1%w/v yeast extract, 1%w/v yeast nitrogen base without amino acids and 1% glycerol) containing glycerol as the sole carbon source, and subsequently transferred to BMMY medium (100 mM potassium phosphate pH 6, 2%w/v peptone, 1%w/v yeast extract, 1%w/v yeast nitrogen base without amino acids and 1% methanol), which induced recombinant protein expression by substitution of glycerol for methanol. After 48 hours of incubation at 28 °C, cell pellets were harvested by centrifugation at 400× *g*. Pellets were resuspended in isolation buffer (50 mM sodium phosphate pH 8.0, 5% glycerol, 1 mM EDTA, and 1 mM DTT) and ruptured via mechanical homogenization using 0.5 mm acid-washed glass beads in a continuous mode DYNO-MILL homogenizer (GlenMills, Clifton, New Jersey, USA). The pH of the obtained lysate was adjusted to pH 8.0 and 2 mM MgSO_4_ was added. After incubation at 4 °C while stirring for 30 min, the supernatant was cleared by centrifugation (30 min at 48,000× *g* at 4 °C) or filtration (0.22 µm Steritop filter). The cleared supernatant was loaded onto a HisTrap IMAC column (GE Healthcare, Chicago, Illinois, USA) equilibrated with isolation buffer and the bound protein was eluted by increasing the imidazole concentration to 400 mM while lowering the NaCl concentration to 20 mM. IMAC-eluted fractions were then pooled for injection on a Superdex75 Hiload 16/600 column (GE Healthcare) equilibrated with FN3K sample buffer (20 mM Tris-HCl pH 8.0, 150 mM NaCl and 1 mM DTT). Fractions containing the protein of interest were pooled, their protein concentration determined spectroscopically and their kinase activity (or lack thereof, for FN3K-K41L) was assayed in vitro using the phosphatase-coupled Universal Kinase Activity Kit (R&D Systems, Minneapolis, Minnesota, USA) with a 1-deoxy-1-morpholino-D-fructose (DMF) acceptor substrate [14]. Briefly, dilution series of FN3K and FN3K–K41L in reaction buffer (25 mM HEPES, 150 mM NaCl, 10 mM MgCl_2_ and 10 mM CaCl_2_, pH 7.0) were incubated with 2 mM DMF in presence or absence of 0.4 mM ATP. After 30 min incubation at 37 °C, phosphatase CD39L2 was added (2 ng/µL) and the release of inorganic phosphate (proportional to the amount of ADP generated during the kinase reaction) was detected at 595 nm using malachite green coloring reagents. After subtraction of negative control (ATP absent) signal, phosphate release as compared to an inorganic phosphate standard curve was quantified for each well. The activity of FN3K and FN3K–K41L was derived from linear regression within the linear range of the dose–response curve. The protein was further characterized on SDS-PAGE and Western blot using either a DyLight800-coupled anti-His antibody or a polyclonal rabbit anti-FN3K antibody (Life Technologies, Carlsbad, California, USA) and a secondary DyLight680-coupled goat anti-rabbit antibody. Purified proteins were aliquoted, snap-frozen in liquid nitrogen, and stored at −80 °C.

### 4.2. Ex Vivo Intravitreal FN3K Injection of Equine Eyes

As a first preliminary experiment, equine (*n* = 3) eye pairs were eviscerated from fresh cadavers and transported to the lab on ice before injection. For each eye pair, one eye was intravitreally injected with 50 µL of an FN3K solution (8.33 µg/mL FN3K, 3.33 mM ATP (Sigma–Aldrich, St. Louis, Missouri, USA), and 1.33 mM MgCl_2_ (Sigma–Aldrich)) and the other eye with 50 µL of a saline solution. Before injection, both eyes of the same animal were evaluated as equally affected by an expert ophthalmologist. After incubation in saline for 24 h at 37 °C, lenses were removed from the intact eye and macroscopic changes of the equine lenses were assessed. In addition, mechanical properties were measured and compared between an FN3K-treated equine lens and its saline-treated counterpart. Mechanical analysis was performed on an LFPlus Universal material tester (Lloyd Materials testing, Bognor Regis, UK) with a ball probe of 4.45 mm in diameter and an indentation speed of 25 mm/min. A lower force limit of 0.1 N was set as the zero-indentation point for the tissue. The tissue was indented by a descending ball probe, to which the central hole was aligned. A load cell of 10 N was used and a maximum force of 5 N was chosen. A five-cycle indentation was performed to exclude the hysteresis effect of the tissue. The stiffness parameter (N/mm), the slope of the linear section of the indentation-load curve, was determined.

### 4.3. Autofluorescence Measurement of AGEs

As described previously [24,45], AGEs were quantified based on Maillard-type autofluorescence (AF) measurements (excitation 365 nm, emission 390–700 nm) using a Flame miniature spectrometer (FLAME-S-VIS-NIR-ES, 350–1000 nm, Ocean Optics, Dunedin, Florida, USA) equipped with a high-power LED light source (365 nm, Ocean Optics) and a reflection probe (QR400-7-VIS-BX, Ocean Optics). Measurements were averaged over 128 scans. AF values were calculated by dividing the average light intensity emitted per nm for the 407 nm to 677 nm range by the average light intensity per nm over the 342 nm to 407 nm range. Analysis of potential interfering agents such as solutions containing ATP (3–100 mM), ADP (12.5 mM, Sigma–Aldrich) and NADPH (10 mM, Sigma–Aldrich) did not reveal notable AF using our setup (data not shown).

### 4.4. FN3K Treatment of AGE-Modified Porcine Lenses

Porcine eyes (*n* = 15) were obtained from a local abattoir and lenses were isolated through dissection by a trained ophthalmologist within 12 h post mortem. The lenses were stored at −20 °C until further processing. Fragments of each lens were added to wells of a black 96-well plate suitable for fluorescence measurements until complete coverage of the bottom was obtained (FluoroNunc PolySorp, Thermo Fisher Scientific, Massachusetts, USA). Fluorescence measurements were executed at baseline for each lens fragment at a fixed distance and straight angle. Since glycolaldehyde is a widely known compound to alter proteins by AGE formation, AGE modification was performed by incubation of the lens fragments with 20 µL of 25 mM glycolaldehyde dimer (crystalline form, Sigma–Aldrich) in phosphate buffered saline (PBS) for 3 h at 37 °C [15]. After incubation, lens fragments were stored for 72 h (4 °C) to ensure completion of the chemical reaction. Subsequently, in vitro deglycation of AGE-modified lens fragments (*n* = 11) was initiated using 20 µL of a solution containing 125 µg/mL FN3K and a fixed amount of ATP (12.5 mM) and MgCl_2_ (5 mM) in phosphate buffered saline (PBS). The remaining lens fragments (*n* = 4) were control treated with 20 µL of a solution containing the cofactors ATP and MgCl_2_ solely. The wells were incubated for 3 hours at 37 °C. Fluorescence measurements were performed immediately after the addition of the FN3K or control solution and repeated after each subsequent hour.

### 4.5. In Vivo Intravitreal FN3K Injection of Murine Eyes

To investigate the effect of FN3K treatment on murine lenses, *ob/ob* mice (*n* = 20, male/female: 8/12, mean age: 24.4 ± 1.4 weeks, BTBR.Cg-Lepob/WiscJ, stock no. 004824, The Jackson Laboratory, Bar Harbor, ME, USA) were anesthetized during surgical procedures with inhalation anesthesia (isoflurane 5%). Both eyes of the same animal were injected with a 32-gauge needle, one with 5 µL FN3K (8.33 µg/mL FN3K, 3.33 mM ATP and 1.33 mM MgCl_2_) and one with 5 µL saline solution. Eyelids were gently moved backwards with a pincet so that injection could be performed just before the equator of the globe with the tip of the needle directed towards the center of the globe, in order not to touch the lens or the retina [46]. Twenty-four hours later, the mice were sacrificed, both eyes were enucleated and the lenses removed. AF measurements were performed on the intact lenses. Afterwards, the geometry of the lenses was evaluated macroscopically. The saline- and FN3K-treated lenses of one mouse were subjected to standard histological examination by a specialized pathologist. Two-micrometer sections were cut, stained with HE, periodic acid Schiff, Jones’ methenamine silver and Masson trichrome, and finally cover-slipped.

### 4.6. Serum Cytokine Levels of FN3K-Treated Mice

*Ob/ob* (*n* = 35, male/female: 16/19, mean age: 26.5 ± 2.9 weeks) and WT (*n* = 20, male/female: 8/12, mean age: 27.6 ± 3.3 weeks) mice were anesthetized and injected intravitreously with 5 µL of the FN3K solution as described above. Using commercial ELISA kits (Ready-SET-Go! ELISA-kits, eBioscience, San Diego, CA, USA), the presence of IL-2, TNFα, and IFNγ was determined in undiluted serum and expressed as pg/mL. Measurements were performed 24 h, 4 days or 7 days after treatment. In addition, cytokine levels in 3 *ob/ob* and 2 WT mice (aged 24 weeks) were measured as negative controls (non-treated).

### 4.7. Human Experiments

#### 4.7.1. Gel Filtration of Human Cataractous Lens Fractions before and after FN3K Treatment

Human lens fragments were obtained following phacoemulsification during cataract surgery from 10 patients. After surgery, the conservation fluid containing the lens fragments was centrifuged (1902× *g*, 5 min, 21 °C) and the supernatant was removed. Afterwards, pooled fragments (20 mg) were incubated for 3 h at 37 °C in 200 µL of a solution containing 125 µg/mL FN3K and a fixed amount of ATP (12.5 mM) and MgCl_2_ (5 mM) in PBS (pH 7.4). Untreated pooled fragments (20 mg) were stored under the same conditions. Since it is well known that cataracts are associated with strong increases in water-insoluble proteins [47], urea-soluble (water-insoluble) proteins from both the untreated and treated lens fragments were extracted in 6 M urea in PBS for 4 h at 4 °C in Eppendorf Safe-Lock Tubes (Hamburg, Germany). After centrifugation (16,000× *g*, 10 min, 21 °C), the supernatant was retained for gel filtration. Gel filtration of untreated and FN3K-treated lens fragments was carried out on a chromatography column (length: 60 cm, diameter: 15 mm) packed with Sephadex G-25^®^ Fine resin (Sigma–Aldrich) in order to assess the molecular mass of fructose containing lens compounds such as AGEs. Following fractionation, individual fractions were photometrically tested using the resorcinol–HCl (Seliwanoff) reaction, a well-known color reaction for ketoses [48]. In this reaction, a 50 µL sample was added to 100 µL resorcinol (9 mM, Sigma–Aldrich) and 1 mL hydrochloric acid (9 M, Sigma–Aldrich). Following incubation in a boiling water bath for 5 min, the developed color was read photometrically in a standard 10 mm cuvette at 488 nm.

#### 4.7.2. Kinetics of FN3K Treatment

Human lens fragments were obtained following phacoemulsification during cataract surgery from 50 patients. After surgery, the conservation fluid containing the lens fragments was centrifuged (3000 rpm, 5 min, 21 °C) and the supernatant was removed. Pooled fragments were added to wells of a black 96-well plate until complete coverage of the bottom was obtained. Subsequently, 20 µL of a solution containing different concentrations of active or mutant FN3K (125 µg/mL, 62.5 µg/mL and 31.8 µg/mL) and a fixed amount of ATP (12.5 mM) and MgCl_2_ (5 mM) in PBS were added to the wells. Besides, a second experiment was performed in a lower concentration range of active FN3K (12.5 µg/mL, 6.25 µg/mL and 1.25 µg/mL). The wells were incubated for 3 h at 37 °C. Fluorescence measurements were performed at baseline and after each subsequent hour. All experiments were performed in triplo.

#### 4.7.3. FN3K Treatment of Intact Eye Lenses

Ex vivo FN3K treatment was performed on the intact left eye lens of a 79-year-old patient with-in the ophthalmological history cataract and an occlusion of the left arteria ophthalmica. The lens from the enucleated human eye was isolated through dissection by a trained ophthalmologist within 24 h post mortem and stored at 4 °C in an RPMI-1640 medium (Thermo Fisher Scientific) until analysis. The experiment was started by removing the RPMI medium and washing the lens with 5 mL PBS solution. Fluorescence measurements were performed at baseline at 15 different measurement locations each on the ventral and dorsal surface of the lens. Subsequently, the whole lens was deglycated by incubation during 20 h at 37 °C in 2 mL of a solution containing 1.6 µg/mL FN3K and a fixed amount of ATP (2.5 mM) and MgCl_2_ (1 mM) in PBS. After incubation, the lens was washed 5 times with PBS and fluorescence measurements were repeated. In addition, an intact cataractous eye lens of an 89-year-old patient was enucleated within 24 h post mortem and deglycated by incubation during 3h at 37 °C in 2 mL of the FN3K solution described above. In addition, a cataractous eye lens originating from another patient was control-treated with a saline solution. Digital images were acquired in a portable LED photocube (Caruba, Beilen, the Netherlands) at baseline and after each subsequent half hour (FN3K treatment) or hour (control treatment) using a Canon EOS 2000D digital camera with EF-S18-55mm f/3.5–5.6 IS II zoom lens (Canon Inc., Tokio, Japan). ImageJ v1.8.0 (NIH, http://rsb.info.nih.gov/ij, accessed on 3 October 2019) was employed for subsequent image analysis.

#### 4.7.4. Topical Treatment of Post-Mortem Eyes

A crossover topical treatment experiment with either saline- or FN3K-containing drops was performed on the enucleated post mortem eyes of a 78-year-old patient with cataractous lenses. The patient’s left eye was treated with saline-containing drops until complete coverage of the eye’s surface was reached. The procedure was repeated after each subsequent hour for 6 consecutive hours. Subsequently, the eye was stored overnight (12 h, 4 °C). Afterwards, a crossover treatment was performed by applying FN3K-containing drops. Again, the procedure was repeated each hour for 6 consecutive hours. The patient’s right eye was processed similarly; however, a reverse order of treatment was applied. AF measurements were performed at a fixed position through the cornea of the intact eyes at baseline, after 6, 18, and 24 h.

### 4.8. Statistical Analysis

Statistical analyses was performed using GraphPad Prism version 8.4.3. (San Diego, CA, USA) Normality of the data was assessed by the Shapiro–Wilk test. Non-normally distributed data are presented as median with the interquartile range (IQR), normally distributed data as mean ± standard deviation (SD). For non-normally distributed data, unpaired differences between 2 groups were assessed using the Mann–Whitney U test and paired differences using the Wilcoxon matched-pairs signed rank test. For normally distributed data, pairwise comparisons between more than 2 groups were accomplished with repeated measures one-way analysis of variance (ANOVA). Subsequently, individual comparisons between 2 groups were performed with paired *t* tests. A *p* value < 0.05 was considered a priori to be statistically significant. 

## 5. Patents

“Compositions for use to treat cataract” patent filed at the European Patent Office. Priority date: 30.01.2018, international application number: PCT/EP2019/051961, international publication number: WO 2019/149648 A1, international publication date: 08.08.2019.

## Figures and Tables

**Table 1:** Maillard-type autofluorescence values (AF values) after crossover topical treatment with a saline and FN3K solution of post-mortem eyes from a 78-year old cataract patient.

Type of Topical Treatment	Time Interval	Measurement Point AF value	AF Value	% Change Compared to Baseline
**Left eye**				
None	Baseline	0 h	0.0166	0
Saline	0–6 h	6 h	0.0162	−2.4
None	6–18 h	18 h	0.0173	+4.2
FN3K	18–24 h	24 h	0.0077	−53.9
**Right eye**				
None	Baseline	0 h	0.0101	0
FN3K	0–6 h	6 h	0.0084	−16.4
None	6–18 h	18 h	0.0079	−21.5
Saline	18–24 h	24 h	0.0086	−14.9

**Figure A1:**
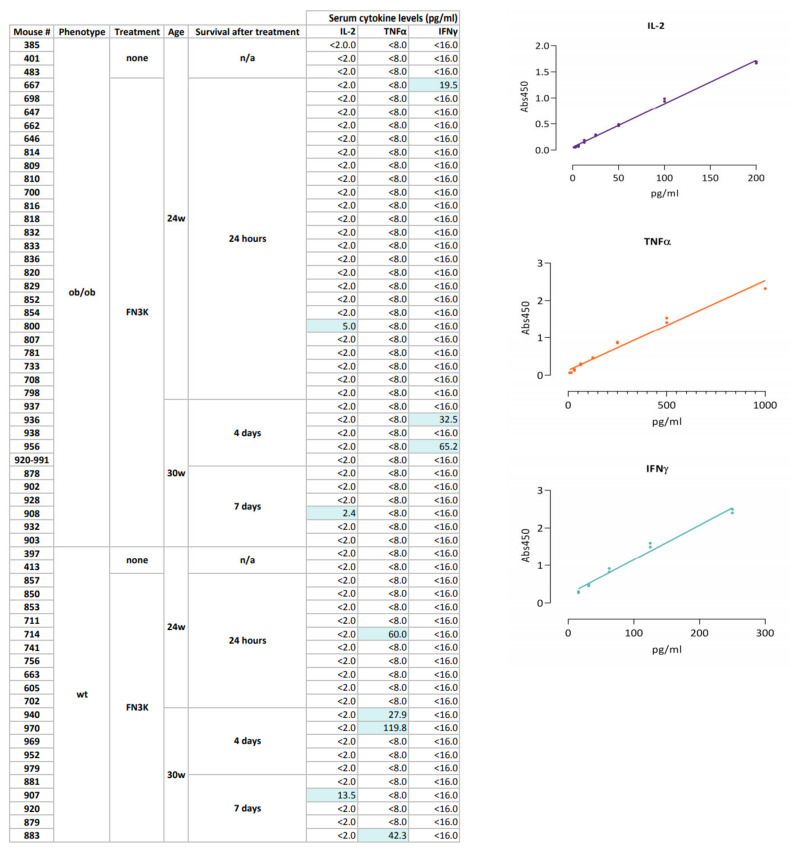
Serum ELISA results of IL-2, TNFα and IFNγ in non-treated *ob/ob* and WT mice (*n* = 5) and FN3K-treated mice (*n* = 55). In all non-treated mice serum levels of IL-2, TNFα and IFNγ were below detection limits (2 pg/mL, 8 pg/mL and 16 pg/mL, respectively). In a large majority of FN3K-treated mice (45 out of 55), levels of these 3 cytokines were below detection limits as well. In several serum samples (10 out of 55), one of the three cytokines tested for was upregulated. However, cytokine levels were not consistently high within treatment groups and in general were very low (below 120 pg/mL). From this, we could conclude that intravitreal injection of a therapeutic FN3K mixture did not consistently provoke a systemic immune response. Calibration curves of IL-2, TNFα and IFNγ are illustrated on the right side.

**Figure A2:**
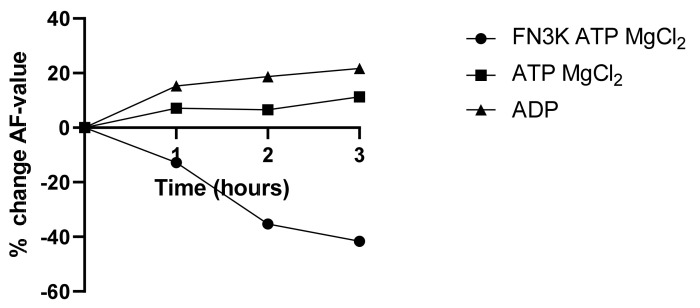
Time-dependent changes (%) in autofluorescence (AF) values after treatment of a cataractous human lens suspension (different suspension as the one used in Section 4.7.2 “Kinetics of FN3K treatment”) with 20 µL of a solution containing fructosamine-3-kinase (FN3K, 125 µg/mL) and its cofactors ATP (12.5 mM) and MgCl_2_ (5 mM) in PBS, (2) the cofactors solely and (3) a solution containing ADP solely (12.5 mM). The experiments were performed in duplicate using the same setup as described in Section 4.7.2. While clear decreases in AF values were observed for the FN3K enzyme, slight increases in AF values were noticed for the solutions containing ATP/MgCl_2_ and ADP. Based on these results, we could conclude that neither the added ATP nor the ADP that was not released by the mutant enzyme was responsible for the observed decreases in autofluorescence after FN3K treatment in our experiments.

## Data Availability

The data that support the findings of this study are available from the corresponding author, upon reasonable request.

## Abbreviations

ADP	Adenosine diphosphate
AF	Autofluorescence
AGEs	Advanced glycation end products
ATP	Adenosine triphosphate
BMMY	Buffered methanol-complex medium
DMF	1-deoxy-1-morpholino-D-fructose
DTT	Dithiothreitol
EDTA	Ethylenediaminetetraacetic acid
ELISA	Enzyme-linked immunosorbent assay
FN3K	Fructosamine-3-kinase
HE	Hematoxylin and eosin
IMAC	Immobilized metal affinity chromatography
IQR	Interquartile range
NADPH	Nicotinamide adenine dinucleotide phosphate
PBS	Phosphate buffered saline
SDS-PAGE	Sodium dodecyl sulphate–polyacrylamide gel electrophoresis
WT	Wild-type
